# HIV-related bilateral inflammatory myofibroblastic tumors of the adrenal gland: a case report and literature review

**DOI:** 10.1186/s12981-022-00492-x

**Published:** 2022-12-24

**Authors:** Mengmeng Zhang, Hui Liu, Zhiqiang Zhu, Yu Zhang, Yanyan Zhang, Xiaopeng Hu

**Affiliations:** 1 Department of Urology, Beijing Youan Hospital, Capital Medical University, Beijing, China; 2 Department of Pathology, Beijing Youan Hospital, Capital Medical University, Beijing, China; 3 Department of Radiology, Beijing Youan Hospital, Capital Medical University, Beijing, China; 4 Department of Urology, Beijing Chao-Yang Hospital, Capital Medical University, Beijing, China

**Keywords:** HIV, Inflammatory myofibroblastic tumor, ART, Adrenal gland

## Abstract

**Background:**

Inflammatory myofibroblastic tumor (IMT) is a rare disease that mostly occurs in younger people and is located in the lungs in the general population. We report a rare case of adrenal IMT in a patient with HIV infection, which is believed to be the first of its kind worldwide.

**Case presentation:**

We present a rare case of a 44-year-old man with HIV infection who was diagnosed with adrenal IMT. The patient refused regular highly active antiretroviral therapy 13 years ago until he was admitted to hospital after an adrenal mass was found. The patient underwent successful computed-tomography-guided needle biopsy, and pathological analysis showed fibroblastic–myofibroblastic proliferation with inflammatory infiltration, which confirmed a diagnosis of IMT. We failed to perform complete resection of the tumor because of its diffuse invasion. The patient was complicated with severe multiple pulmonary infections postoperatively because of immunodeficiency, which eventually caused his death 2 months later.

**Conclusion:**

Differential diagnosis of IMT is difficult, and tumor biopsy is an essential means of diagnosis. Surgical resection is preferred for both adrenal and HIV-related IMTs. Conservative treatment should be considered when there are technical difficulties with complete resection, and most patients have achieved good outcomes. However, more cases and longer follow-up are warranted to confirm long-term outcomes of HIV-related IMT.

## Background

Inflammatory myofibroblastic tumor (IMT) originates from mesenchymal tissue and is a rare disease worldwide. IMT has been referred to as inflammatory pseudotumor, pseudosarcomatous myofibroblastic proliferation, inflammatory sarcoma, plasma cell granuloma, and inflammatory myohistocytic proliferation [1]. IMT mostly occurs in younger people and is located in the lungs in the general population, although other less common sites have gradually been reported in recent years, such as liver, pancreas, pharynx, spinal canal, and retroperitoneal space [1–4]. Nowadays, we are used to thinking of IMT as a low-grade malignant tumor with pathological features of proliferation of fibroblastic–myofibroblastic cells with inflammatory infiltration, and the potential of local recurrence but a low risk of distant metastases [5].

Infection with the human immunodeficiency virus (HIV) results in progressive loss of immune function marked by depletion of the CD4 + T-lymphocytes, leading to opportunistic infections and AIDS-defining cancers such as Kaposi’s sarcoma, non-Hodgkin lymphoma, and invasive cervical carcinoma [6]. However, the increased incidence of non-AIDS-defining cancers has been accompanied by improved life expectancy because of the advent of antiretroviral therapy (ART) worldwide [7]. IMT is a type of non-AIDS-defining tumor that has only been reported in a few patients with HIV infection [8–17]. We present the first case worldwide of HIV-related IMT located in the adrenal gland, and a review of the literature on HIV-related IMTs and adrenal IMTs.

## Case presentation

**Fig. 1:**
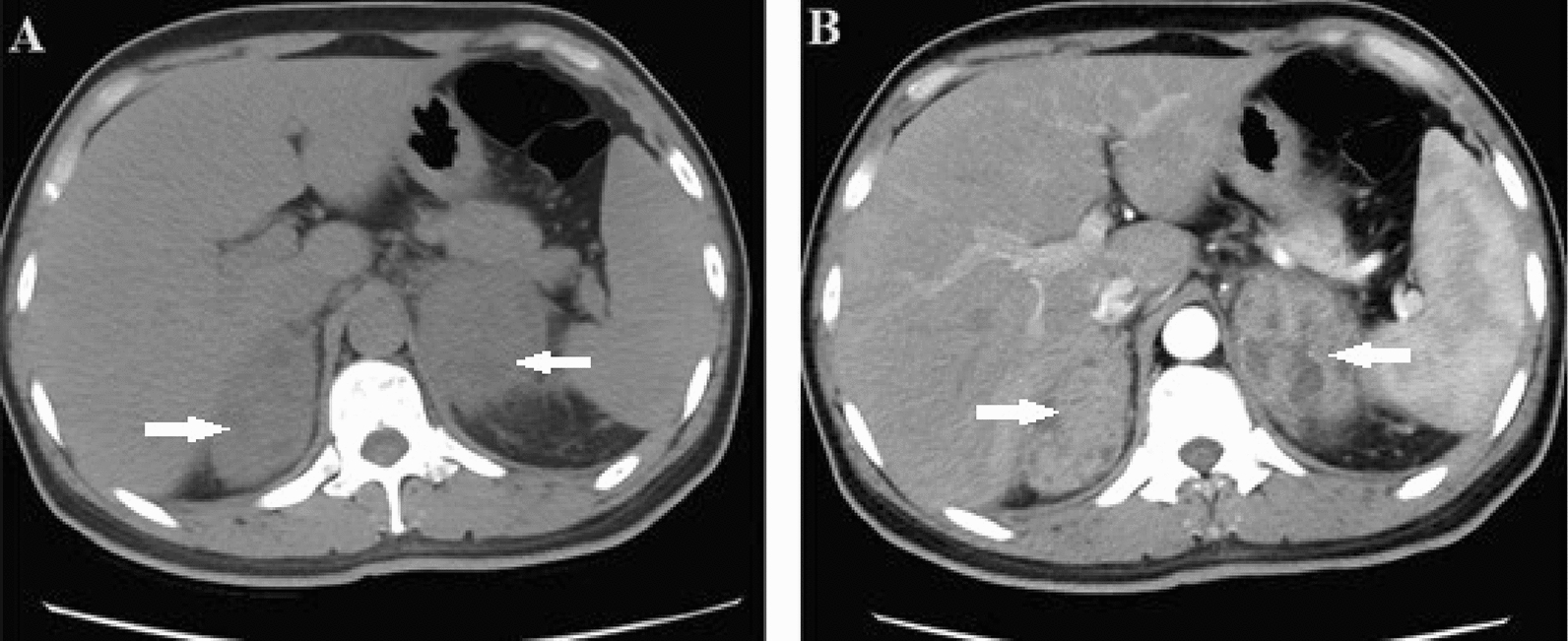
** A** Transverse view of unenhanced computed tomography (CT) showed hypodense and heterogeneous solid lesions adjacent to both adrenal glands. **B** Transverse view of Enhanced CT showed moderate and heterogeneous enhancement of masses

**Fig. 2:**
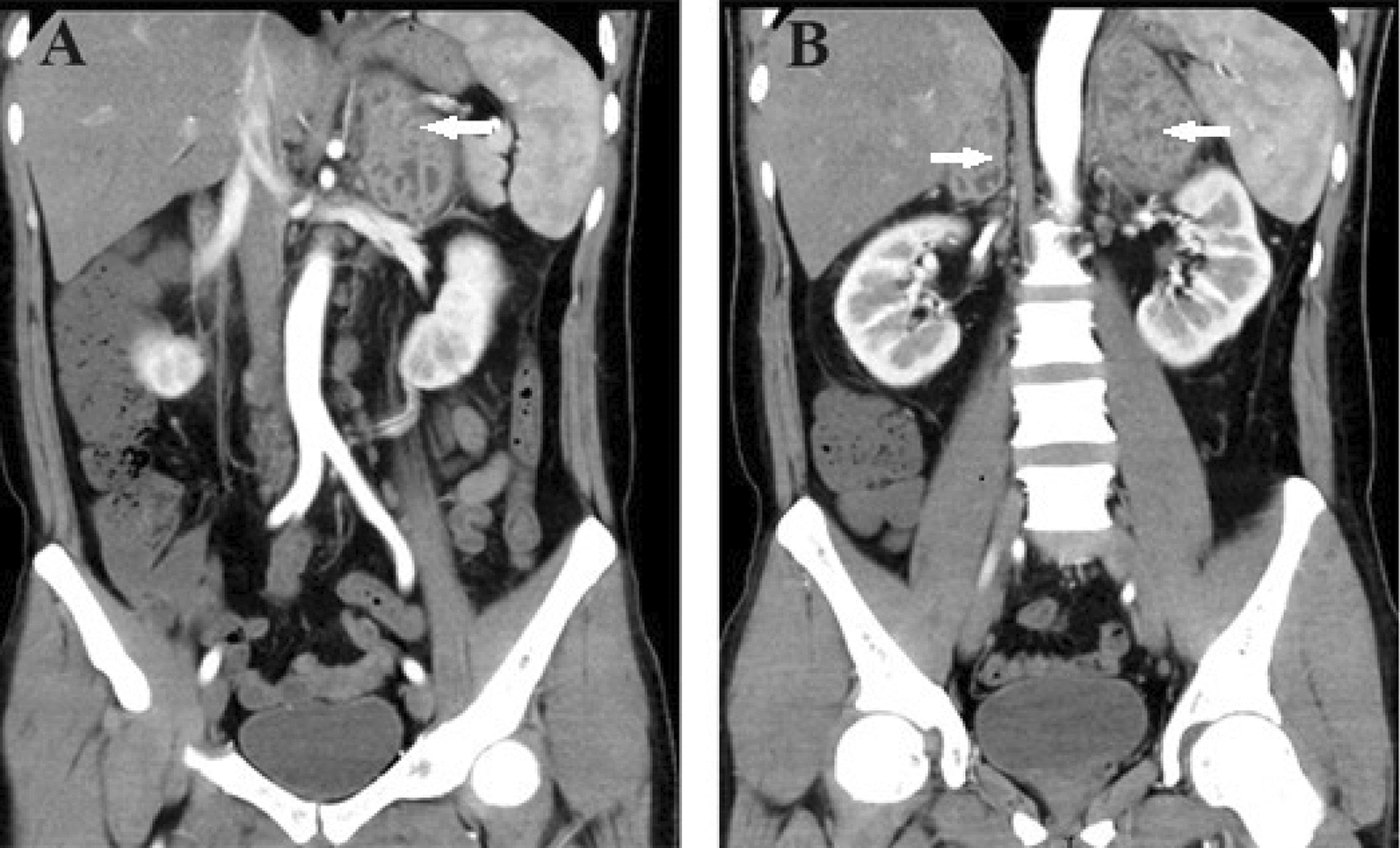
Coronal view of enhanced computed tomography.** A** Enlargement of lymph nodes around the aorta. **B** Inability to recognize normal anatomical structures of the adrenal glands because of tumor compression

A 44-year-old man presented with a 3-week history of persistent dull back pain, accompanied by fever, fatigue, and weight loss. The patient refused to accept ART after diagnosis of HIV infection 13 years ago. There was a tapping pain at the costovertebral angle, and no other positive findings on physical examination. The decreased CD4^+^ T-lymphocyte count (6 cells/µL) and increased HIV load (10,001,391 copies/mL) indicated advanced immunodeficiency; however, laboratory examination showed no evidence of opportunistic infection with cytomegalovirus, Epstein–Barr virus, fungi, or tuberculosis. In terms of fungal infections, the serum (1,3)-β-D-glucan test, the galactomannan test and the fungal blood culture were normal or negative, mainly to detect Aspergillus and Candida infections. The haematological and biochemical examinations indicated mild anemia (103 g/l), elevation of aspartate transaminase (79u/l) and gamma-glutamyltransferase (207u/l), and hypoalbuminemia (30.7 g/l), and other results were normal. A variety of hormones including renin, angiotensin, aldosterone, adrenocorticotropic hormone, cortisol, and catecholamine were within normal range, and is considered to be associated with functional adenomas in the adrenal area. Abdominal unenhanced computed tomography (CT) showed two hypodense and heterogeneous solid lesions adjacent to both adrenal glands; the left lesion was approximately 67⋅38 mm and the right lesion was approximately 56⋅58 mm (Fig. 1A). Enhanced CT showed moderate and heterogeneous enhancement (Fig. 1B), following enlargement of lymph nodes in the retroperitoneal space, accompanied by an inability to recognize the normal anatomical structures of the adrenal glands because of tumor compression (Fig. 2A, B). We initially considered diagnosis of lymphoma in light of the imaging features and severe immunodeficiency caused by HIV. Adrenal malignancy and pheochromocytoma were not excluded, and CT-guided biopsy of the lesion adjacent to the right adrenal gland was performed.

**Fig. 3:**
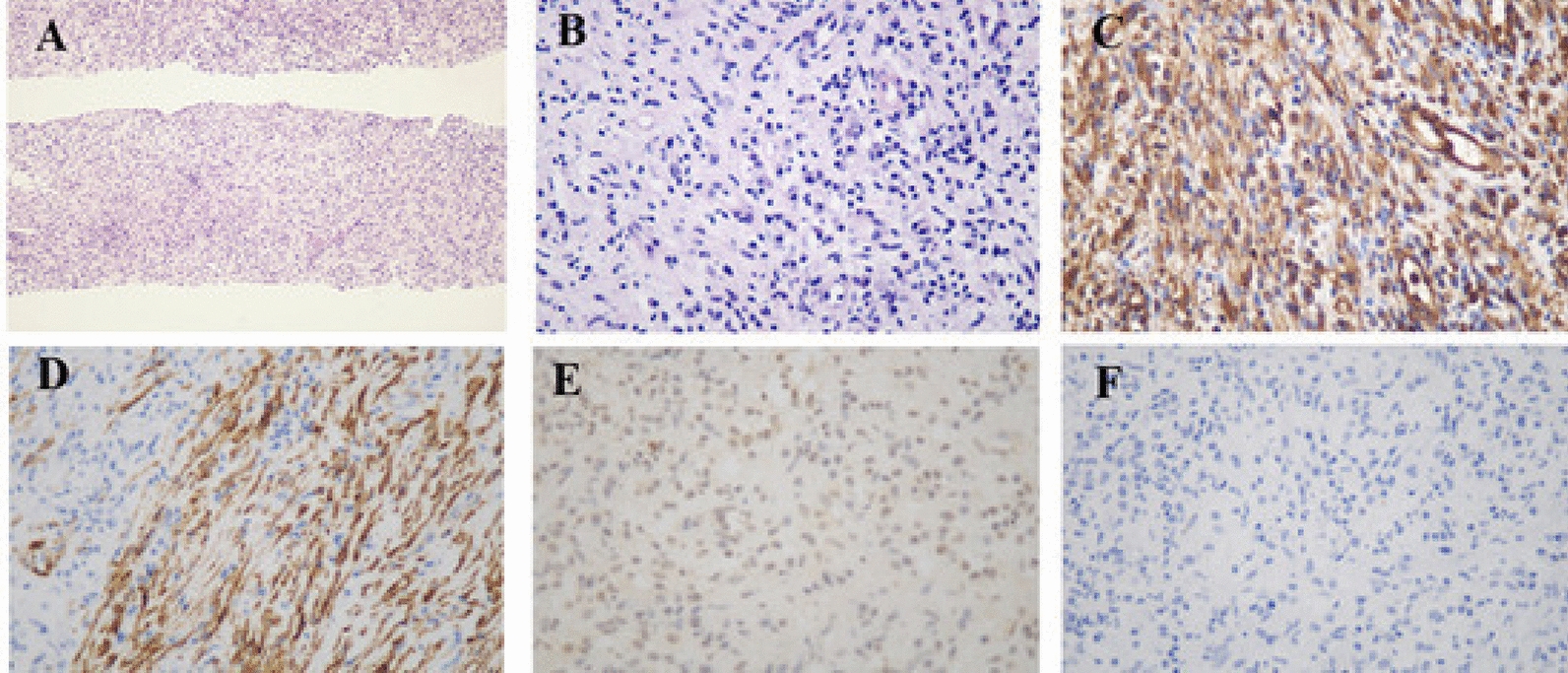
Histological and immunohistochemical findings. **A** hematoxylin and eosin (HE), 100×. **B** HE, 400×. **C** Diffusely and strongly positive for vimentin. **D** Focally and weakly positive for smooth muscle actin. **E** Negative for anaplastic lymphoma kinase 1. **F** Negative for S100

The pathological analysis of biopsy specimens confirmed the diagnosis of IMT. The specimen measured 4⋅8⋅12 mm, 1 mm in diameter, with a gray–white surface. Histological findings revealed proliferation of spindle cells without obvious atypia and small vessels in a myxoid and collagenous background with infiltration of plasma cells, lymphocytes, and neutrophils. Collagenous sclerosis and myxoid degeneration were seen in some areas. The spindle cells did not exhibit nuclear pleomorphism (Fig. 3A, B). Immunohistochemical staining confirmed that these spindle cells were diffusely and strongly positive for vimentin (Fig. 3C), and focally and weakly positive for smooth muscle actin (Fig. 3D). Anaplastic lymphoma kinase (ALK)1 and S-100 protein were negative (Fig. 3E, F). IgG4, CD34, and CD38 immunostaining was also focally positive.

**Fig. 4:**
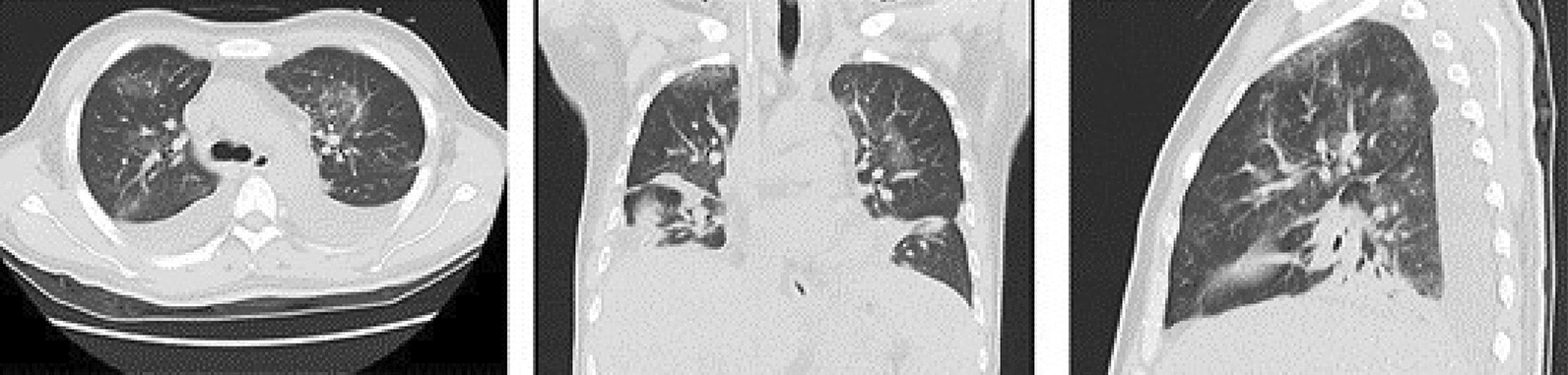
Pulmonary computed tomography showed extensive inflammatory lesions in both lungs

We used emtricitabine (200 mg once daily), tenofovir disoproxil fumarate (300 mg once daily), and dolutegravir (50 mg once daily) to control HIV infection, and trimethoprim-sulfamethoxazole was given prophylactically after admission. After 1 month of ART, CD4^+^ T-lymphocyte count increased to 33 cells/µL; however, back pain became progressively worse. As a result of the uncertain efficacy and adverse effects of chemotherapy, steroid therapy, and radiotherapy for HIV infection, we planned to perform staging laparoscopic resection of bilateral lesions. Complete resection was not possible, due to extensive invasion into surrounding organs (pancreas, kidney, and diaphragm) and undefined margins. The patient’s postoperative course was complicated by hospital acquired pneumonia (Fig. 4), which made adjuvant therapy impossible. However, no organisms were isolated, regardless of blood or sputum. The patient died of sepsis caused by pulmonary infection 2 months after surgery.

## Discussion and conclusion

On the basis of this rare case report of HIV-related IMT arising in the adrenal glands, we conducted a literature search on PUBMED using the terms “Inflammatory myofibroblastic tumor,” “Inflammatory pseudotumor,” “Pseudosarcomatous myofibroblastic proliferation,” “Inflammatory sarcoma,” “Plasma cell granuloma,” “Inflammatory myohistocytic proliferation,” “Human immunodeficiency virus,” and “Adrenal gland” and identified 10 papers describing 10 cases of HIV-related IMT (Table 1) and 10 papers involving 10 cases of adrenal IMTs (Table 2).

**Table 1:** Previous case reports of HIV-related IMTs

Author	Country, year	Age, Sex	CD4^+^ T-cells (cells/ul)	Viral load (copies/mL)	ART duration (months)	Location, presentation	Treatments	Follow-up duration, outcomes
Carlos A [8]	USA, 1995	23y, F	NA	NA	NA	Larynx, dyspnea	No	Sudden death
De Castro [9]	USA, 2000	43y, M	185	741	30	Retroperiton- eum, pain	Thalidomide	1 year, NR
Vaideeswar [10]	India, 2000	32y, M	NA	NA	NA	Submandibu- lar, swelling	NA	NA
Braun [11]	Spain, 2003	38y, F	91	213,000	12	Spleen, pain	spleenectomy	10 months, NR
Chan-Tack [12]	USA, 2006	26y, M	142	79	5	Sinuses, pain and swelling	Anterior ethmoidectomy	3 months, NR
Mazhari M [13]	UK, 2006	27y, M	NA	NA	NA	Testicle, lump and pain	radical right orchidectomy	NA
Liu [14]	China, 2011	20y, M	272	TND	60	Right thigh, swelling	right hip joint amputation	10 months, SFE
Cambrea [15]	Romnia, 2014	21y, M	23	High	144	Lung, respirat-ory symptoms	antibiotics, antiviral, antifungal	1 year, asymptomatic and SFE
Ramotar [16]	UK, 2015	49y, F	500	< 50	NA	Head and neck, lump and odynophagia	Surgical resection	NA, asymptom-atic and NR
Bai [17]	USA, 2020	52y, M	NA	NA	NA	Rectum, abdo-minal pain	valacyclovir	asymptomatic and NR

*F* female,* ART* antiretroviral therapy,* IMT* inflammatory myofibroblastic tumor,* M* male,* NA* not available,* NR* no recurrence,* SFE* significantly favorable evolution,* TND* target not detected.

**Table 2:** Previous case reports of adrenal IMTs

Authors	Year	Age	Sex	Presentation	Treatments	Laterality	Maximum dimension	Follow-up duration/ Outcomes
Mascarel [18]	1989	17y	F	Secondary amenorrhea	Adrenalectomy	Left	10 cm	2 years, NR
Luo [19]	2006	2y	F	Fever	Tumor resection	Right	5 cm	Half year, NR
Fragoso [20]	2011	28y	F	Palpable mass	Nephroadrenalectomy	Isolateral	7 cm	13 years, NR
Wang [21]	2011	57y	F	Incidental findings	Therapeutic, laparotomy	Left	8.5 cm	NA
Chawla [22]	2013	20y	M	Flank pain	Tumor resection	Left	7 cm	3 months, NR
Tran-Dang [23]	2014	29y	M	Flank discomfort	Adrenalectomy	Right	17.2 cm	NA
Xu [24]	2015	35y	F	Abdominal pain, fever	Tumor resection, adjuvant NSAIDs	Multiple/Left	4.5 cm	1 year, NP
Chen [25]	2016	60y	M	Abdominal pain	Radiofrequency ablation, chemotherapy	Left	6.7 cm	NP
Sannaa [26]	2016	34y	M	Back pain	Adrenalectomy	Right	11 cm	4 years, NR
Zhang[27]	2017	56y	M	Fatigue	Tumor resection	Right	12 cm	NA

*F* female,* IMT* inflammatory myofibroblastic tumor,* M* male,* NA* not available,* NR* no recurrence,* NP* no progression.

Previous studies have shown that the most common site of IMT is the lungs, while the adrenal gland is an unusual location [18]. However, only one case of HIV-related IMT occurred in the lungs, and its distribution was scattered over different sites, which is obviously different from the general population [8–17]. Our patient presented with back pain accompanied by other manifestations such as fever, fatigue, and emaciation, which was similar to former reports of adrenal IMT [19–27]. Additionally, CT showed bilateral masses with no specifically enhanced images. Above all, the patient had a 13-year history of HIV infection with no treatment, which had resulted in advanced immunodeficiency. In light of the above features, AIDS-defining cancers were given priority in the differential diagnosis, such as lymphoma and Kaposi’s sarcoma. However, the differential diagnosis still included adrenal diseases that occurred frequently in the general population, such as functional adrenal adenoma, adrenal cortical carcinoma, and adrenal metastases [18–27]. Differential diagnosis is sometimes difficult in the absence of typical symptoms. CT-guided biopsy of the lesion is necessary to make a diagnosis of IMT if feasible.

The diagnosis of IMT ultimately depends on histopathological analysis. In our case, pathological analysis revealed proliferation of spindle cells and infiltration of plasma cells, lymphocytes, and neutrophils, in line with features reported previously [28, 29]. An immunohistochemical study showed that ALK1 was negative, which indicated less aggressive pathology and less likelihood of recurrence [30]. A study of 84 cases suggested a 25% increase in the recurrence rate for ALK1-positive compared with ALK1-negative IMT specimens [31]. However intraoperative findings showed more aggressive pathological features in our case, which was inconsistent with the predictive effect of ALK. This contradiction might be associated with immunodeficiency caused by HIV infection.

The etiology of IMT is still unknown and disputable. Some experts believe that an over-reaction to infection or trauma is at the root of IMT [20]. As early as 1995, the frequent presence of Epstein-Barr virus in IMT has been documented. Human papillomavirus and *Helicobacter pylori* have also been confirmed in IMT tissues, but the specific mechanism remains to be fully investigated, which may be relevant to the cytokine release and B-lymphocyte differentiation caused by infection [11, 20]. The etiology of HIV-related IMT should also include the above mechanism, given that immunostaining in recent cases has demonstrated herpes simplex virus infection in rectal IMT [17]. Some experts believe that the occurrence of HIV-related IMT has a potential relationship with immune reconstitution inflammatory syndrome (IRIS), which is characterized by a paradoxical clinical worsening under the background of an improving immune system. There was a case of IMT presenting as sinusitis that occurred 20 weeks after starting ART, and the author suggested that there might be a link between IMT and IRIS[12]. Most cases of IRIS occur within 8 weeks of initiation of ART and develop among patients with CD4 T-lymphocyte counts < 100 cells/µl; however, a few cases occur several years after reconstitution of the immune system [12]. We did not consider the association with IRIS because ART had not been started at the time of tumor detection and advanced immunodeficiency, but IRIS might partly explain the worsening pain the patient experienced after commencing ART.

Complete surgical resection is the main treatment for most IMTs, and mass biopsy is recommended to avoid organ resection in patients in whom it is difficult to differentiate IMT from primary organ malignancy. A retrospective study of 22 urinary IMTs that were treated with complete tumor resection or radical organ resection documented good outcomes with no recurrence or metastases after a median follow-up of 6.1 years [32]. If surgical resection is technically difficult, conservative treatments including steroid therapy, antibiotics, radiotherapy, vinorelbine and methotrexate combination chemotherapy, or carbon dioxide laser should be attempted. A recent study showed that ALK inhibitors appeared to be beneficial as adjuvant therapy for ALK-positive IMT, and they also have proven efficacy for ALK-positive non-small cell lung cancer [33]. In a multicenter prospective study, 12 patients with IMT achieved an objective response of 50% after adjuvant therapy with ALK inhibitor crizotinib [34]. ALK inhibitors are a type of targeted therapy that are effective for IMT with incomplete resection. We did not succeed in mass resection because of undefined margins and extensive invasion of the tumor that might have been caused by immunodeficiency. We did not recommend any adjuvant therapy because of the complication of uncontrolled pulmonary infection that might have been caused by endotracheal intubation during anesthesia. In previous studies of HIV-related IMT, some cases underwent successful mass resection [11–14, 16], while the remaining cases had the same conservative treatment as the general population [9, 15, 17].

IMT used to be considered a benign tumor since it was first reported in the lungs in 1939 [35]. Most IMTs have had good outcomes mainly due to low degree of malignant potential, low rates of recurrence and distant metastasis (2–25% with recurrence, less than 5% with metastasis), and high probability of complete surgical resection [4, 32]. Pathologists have now found kinds of cytogenetic alterations in different cases involving ROS1, PDGFRβ, NTRK3, and RET, suggesting tumors’ malignant potential [36, 37]. Nevertheless, the outcome of IMT is uncertain when it is related to HIV infection because there are only a few relevant case reports. To our knowledge, only 10 cases have been reported globally; most of which seemed to have good outcomes after follow-up of 3 months to 1 year [9–17]. In our case, the patient died of severe pulmonary infection within 2 months of unsuccessful surgery that made it impossible to try adjuvant therapies and track the natural course of the tumor, which was aggravated by the advanced HIV infection. More cases and longer follow-up are warranted to confirm the outcomes of HIV-related IMT.

HIV-related IMTs appear to be more likely to occur in extrapulmonary sites. Differential diagnosis of IMT is difficult owing to the lack of specific clinical manifestations and imaging features, and tumor biopsy should be utilized as an essential means of diagnosis. The etiology of IMT remains uncertain and may be related to IRIS in HIV-positive patients. Surgical resection is preferred for both adrenal and HIV-related IMTs. Conservative treatment should be considered when there are technical difficulties with complete resection, and most patients have achieved good outcomes. Our case reminds physicians that patients with HIV-related IMT with advanced immunodeficiency might have a poor prognosis. However, more cases and longer follow-up are warranted to confirm long-term outcomes of HIV-related IMTs.

## Data Availability

The datasets used and analyzed during the current study are available from. the corresponding author on reasonable request.

## Abbreviations

IMT: inflammatory myofibroblastic tumor
ART: antiretroviral therapy
CT: computed tomography
SMA: smooth muscle actin
ALK1: anaplastic lymphoma kinase 1
IRIS: immune reconstitution inflammatory syndrome.
